# Two Cases of Q-Fever in Hairy Cell Leukemia

**DOI:** 10.1155/2014/863932

**Published:** 2014-08-12

**Authors:** Emanuele Ammatuna, Emilio Iannitto, Lidwine W. Tick, Nicolaas L. A. Arents, Philip H. Kuijper, Marten R. Nijziel

**Affiliations:** ^1^Department of Hematology, Erasmus University Medical Centre, Daniel den Hoed, Groene Hilledijk 301, 3075 EA Rotterdam, The Netherlands; ^2^Department of Oncology, Haematology Unit, University of Palermo School of Medicine, Palermo, Italy; ^3^Department of Internal Medicine, Maxima Medical Centre, The Netherlands; ^4^Laboratory for Medical Microbiology, PAMM Foundation, Veldhoven, The Netherlands; ^5^Department of Clinical Chemistry, Máxima Medical Centre, Veldhoven, The Netherlands

## Abstract

Hairy cell leukemia (HCL) is a rare B-cell lymphoproliferative disorder accounting for about 2% of all leukemias. The clinical course is indolent, however HCL patients are particularly susceptible to infections. Here we report two cases of Q-fever as first manifestation of disease in two patients affected by HCL. Both patients described in this report showed an unusually sluggish clinical response to the antibiotic treatment with ciprofloxacin probably because of the marked immunodeficiency. However, treatment of HCL with cladribine administered soon after the resolution of QF pneumonitis was uneventful and led to a complete remission in both cases. Most probably the association of Coxiella burnetii (CB) infection and HCL that we observed in two patients is due to chance. However, a hairy cell resembling transformation of freshly isolated human peripheral blood lymphocytes upon CB has been showed. We think that the possibility of CB infection in febrile HCL patient should be always taken in mind, especially in endemic areas. In addition the potential for such infections to become chronic in HCL patients should not be overlooked and the reporting of further cases should be encouraged.

## 1. Introduction

Hairy cell leukemia (HCL) is a rare B-cell lymphoproliferative disorder accounting for about 2% of all leukemias [1]. The median age of diagnosis is 56 years, and it affects mainly elderly men, with a male to female ratio of 4 : 1 [2]. The neoplastic clone usually grows slowly and homes preferentially in bone marrow and spleen with a subtle spill-over in the peripheral blood resulting in a clinical picture characterized mostly by splenomegaly and pancytopenia. The clinical course is quite indolent; however, the peculiar clinical characteristic of patients affected by HCL is the susceptibility to develop severe life threatening infections. Approximately 25% present with infection and 70% have either documented or suspected infection during the course of the disease. The susceptibility to infection is higher in those patients with active disease and high tumor burden, while it is markedly reduced in those in complete remission after effective treatment. Fifty percent of these infections comprise those typically seen in a neutropenic host. The rest of reported cases include atypical mycobacterial infections,* Listeria*,* Pneumocystis*,* Legionella*, viral infections, Aspergillus, and other fungal infections [3, 4].

Q-fever (QF) is a worldwide zoonosis caused by* Coxiella burnetii* (CB). CB is a gram-negative and obligate intracellular bacterium that tends to infect mononuclear phagocytes but can infect other cell types as well. The course of human infection ranges from asymptomatic to severe but typically results in a mild, self-limiting, influenza-like disease making the diagnosis challenging and probably under reported [5, 6]. To date, only one case of QF in HCL patient has been described [7]. Here we report two patients with severe Q-fever as an initial manifestation of HCL.

## 2. Cases Presentation

### 2.1. Case  1

In March 2009, a 48-year-old man was admitted to the ward following one week of high fever, chills, cough, dyspnea, and disorientation. His past medical history was unremarkable and he did not report any contact with animals such as cattle or parrots but he was used to taking a daily walk passing a petting zoo. Physical examination revealed a temperature of 39.6°C, a respiratory rate of 32/minute, and rhonchi and crackling sounds at right lung base. The spleen was felt 2 cm under the costal margin and no enlarged superficial lymph nodes were detected. Blood and sputum culture were negative for growth of fungi and gram+ or gram− bacteria. Hemoglobin level was 7.2 mmol/L, platelet count was 83 × 10^9^/L, and the total WBC count was 1.1 × 10^9^/L. The differential leukocyte count showed neutropenia (0.5 × 10^9^/L) and lymphopenia (0.6 × 10^9^/L). A chest X-ray showed a consolidation in the right basal lobe. The patient was empirically treated with intravenous penicillin 6 million international units/day and oral ciprofloxacin 500 mg b.i.d. for 7 days. However, after a week of antibiotic treatment neither the clinical picture nor the marked pancytopenia showed any improvement. Bone marrow trephine biopsy and bronchoscopy with bronchus brushing were performed. The bone marrow histological analysis revealed a heavy interstitial infiltration of lymphoid cells with a typical fried egg pattern. Immunophenotypic analysis of the bone marrow aspirate showed the following: CD20++ CD22+, CD103+ CD25+/− CD11c+/− CD10−CD23−CD5− and the diagnosis of HCL was made. The bronchoscopy with bronchus brushing (BAL) revealed a positive CB PCR which led to the diagnosis of QF and prompted an antibiotic switch to doxycycline monotherapy. Because of an allergic reaction characterized by diffuse rash covering the entire body surface, he was further treated with oral ciprofloxacin monotherapy. Clinical conditions slowly ameliorated and all signs and symptoms of pneumonitis regressed except for the fever and the marked pancytopenia. After an extensive search for other infective causes, the persistent febrile status was interpreted as tumor related and 3 weeks after the admission the patient was treated with cladribine 0.15 mg/kg for 5 consecutive days for his primary hematological disease. The clinical course was uneventful and the patient achieved a complete remission (CR) and resolution of all symptoms. At the 4-year follow-up the patient is still in CR and the CB DNA in the peripheral blood is undetectable.

### 2.2. Case  2

In May 2009, a 56-year-old man was seen in the emergency room because of fever, chest pain, and dyspnoea accompanying atrial fibrillation. Prior to admission he was prescribed amoxicillin/clavulanic acid t.i.d. during 4 days because of a pneumonic process in the medium lobe. His past medical history was unremarkable and he did not report any recent travel outside of The Netherlands nor contact with animals. At admission, physical examination revealed a body temperature of 40.0°C, respiratory rate of 36/min, and crackling sounds at medium and lower right lung lobe. The spleen was felt 4 cm under the costal margin but no enlarged superficial lymph nodes were identified. Laboratory analysis showed a severe pancytopenia (hemoglobin: 4.6 mmol/L, leukocytes 1.1 × 10^9^/L, and thrombocytes 23 × 10^9^/L). Blood and sputum culture were negative for growth of fungi and/or gram+ or gram− bacteria. The patient was empirically treated with penicillin 6 million international units/day and oral ciprofloxacin 500 mg b.i.d. Five days after admission, the clinical condition worsened and the patient developed respiratory failure that necessitated mechanical ventilation at the intensive care unit. At this point antibiotics were switched to intravenous ceftazidime 1500 mg t.i.d. and vancomycin 1 gr b.i.d. A peripheral blood immunophenotype assay was performed to investigate a possible underlying blood disease. This revealed a monoclonal B-cell population CD20++ CD22+, CD25+ CD103+− CD11c+ CD10−CD23−CD5− compatible with the diagnosis of HCL. Clinical conditions and fever did not improve and the patient underwent a BAL and successively a wedge excision of the medium lobe. Both procedures did not show any fungal or bacterial infection. Subsequently, an acute renal failure due to acute tubular necrosis requiring dialysis developed. At that point, a rising phase 2 antibody titre against CB was detected and the diagnosis of QF was made. Treatment with doxycycline was started but because of liver toxicity was switched to ciprofloxacin. Moreover, chemotherapy with cladribine 0.15 mg/kg for 5 consecutive days for the treatment of the HCL was delivered. After a few weeks, clinical conditions ameliorated and the patient was discharged from the intensive care unit following complete recovery from pneumonia and renal failure. While asymptomatic, for the persistent positive CB PCR in the peripheral blood, treatment with daily moxifloxacin 400 mg and plaquenil 200 mg t.i.d. for 18 months was carried out. During this period the CB PCR assay became negative and the phase 2 IgG antibody titer reduced consistently. Four years after cladribine treatment, the patient is still in complete remission from his HCL.

## 3. Discussion and Conclusion

The most common clinical manifestation of acute QF is a nonspecific febrile illness followed by pneumonia and hepatitis. However, asymptomatic infections have been reported in up to 60% of cases identified during outbreak investigations. This fact indicates that other factors than CB exposure are responsible for the severity of this disease. Our patients suffered from a severe form of acute QF leading to a respiratory insufficiency. This can be explained by the severe immunocompromised status typical of patients affected by HCL. Indeed, poor granulocyte reserve and mobilization and several defects of innate and acquired immunity as well as the marked monocytopenia and functional abnormalities of monocytes and dendritic cells make HCL patients particularly vulnerable to infections caused by intracellular microorganisms [8–10]. The risk is particularly high at disease onset and during the early treatment period due to the neutropenia and immunosuppression caused by the purine analogues.

The first and most obvious consideration from the analysis of these cases is to consider the possibility of QF as a cause of pneumonitis in patients with HCL, particularly in an endemic area or in patients belonging to high occupational risk groups with the latter being workers in slaughterhouses, farms, and animal research facilities. We would also underscore that in both patients described in this report, QF showed an unusually sluggish clinical response to the antibiotic treatment with ciprofloxacin probably because of the marked immunodeficiency. However, treatment of HCL with cladribine administered soon after the resolution of QF pneumonitis was uneventful and led to a complete remission in both cases. Prior to this paper, only one case of CB infection associated with HCL has been reported in the literature. Most probably the association of CB infection and HCL that we observed in two patients is due to chance in an endemic area for CB such as the North Brabant province in The Netherlands. However, a very interesting observation was done by Lee and coworkers, who showed that a hairy cell resembling transformation of freshly isolated human peripheral blood lymphocytes (PBL) could be observed in vitro upon CB infection [11]. Moreover, in two-thirds of cases, the transformed PBL acquired tartrate resistant phosphates acid expression, a phenotype quite distinctive of HCL. We certainly acknowledge that these data, although fascinating, are insufficient to speculate on a possible role of CB in the developing of HCL. In conclusion, we think that the possibility of CB infection in a febrile HCL patient should be always taken in mind, especially in endemic areas. In addition the potential for such infections to become chronic in HCL patients should not be overlooked and the reporting of further cases should be encouraged.
